# Optical Sensor Array for the Early Diagnosis of Alzheimer’s Disease

**DOI:** 10.3389/fchem.2022.874864

**Published:** 2022-04-04

**Authors:** Fei Li, Callum Stewart, Shijie Yang, Fangfang Shi, Wenyu Cui, Shuming Zhang, Hao Wang, Hui Huang, Mingqi Chen, Jinsong Han

**Affiliations:** ^1^ State Key Laboratory of Natural Medicines, Department of Food Quality and Safety, National R&D Center for Chinese Herbal Medicine Processing, College of Engineering, China Pharmaceutical University, Nanjing, China; ^2^ Wai Lau Centre for Reparative Medicine, Karolinska Institutet, Hong Kong, China

**Keywords:** sensor array, alzheimer’s disease, chemical tongue, fluorescent, colorimetric, biomarker, pattern recognition, machine learning algorithm

## Abstract

Alzheimer’s disease (AD) is the most common neurodegenerative disorder and has complicated pathobiology, leading to irreversible memory loss and severe cognitive dysfunction. For patients with AD, the advent of the disease usually occurs after years of pathological changes. The early diagnosis and monitoring of AD are of great significance as the early-stage intervention and treatment may be the most effective. Biomarkers, such as beta-amyloid and tau levels in cerebrospinal fluid (CSF) and brain, offer one of the most promising paths and are combined with neuroimaging and immunological detection for AD diagnosis. However, high expense and radiation of neuroimaging and low sensitivity of immunosorbent assay limited their applications. Meanwhile, the relevance of A*β* peptides and tau proteins to the development of AD remains highly debatable, meaning that detecting one specific biomarker holds limited prospects in achieving early and accurate detection of AD. Optical sensor arrays based on pattern recognition enable the discrimination of multiple analytes in complicated environments and are thus highly advantageous for the detection of AD with multi-biomarkers. In this review, we survey the recent advances of optical sensor arrays for the diagnosis of AD, as well as the remaining challenges.

## Introduction

Dementia is defined as the decline in internal patterns of memory and thinking involving at least two cognitive or behavioral impairments (Long and Holtzman, 2019; McKhann et al., 2011). Alzheimer’s disease (AD) has been the most common cause of dementia in older adults, accounting for 60–80% of dementia cases. AD is characterized by the accumulation of fibrillar amyloid *β* (A*β*) peptides in extracellular plaques and hyperphosphorylated tau protein aggregates in intracellular neurofibrillary tangles (Knopman et al., 2021). These pathological changes usually occur several years before clinical symptoms manifest in patients. The identification and easy detection of biomarkers signaling the early stages/window of AD development has recently emerged and opens the opportunity for early-stage interventions.

The conventional detection techniques of AD biomarkers (A*β* peptides and tau proteins) mainly rely on neuroimaging and immunological assays (Ariza et al., 2015; Tago et al., 2016; Huang et al., 2021). However, the high cost and radiation exposure of positron emission tomography imaging, as well as complicated and time-consuming operations of low sensitivity immunosorbent assays, limited their real-life applications. Consequently, simple and rapid detection methods of AD are urgently required.

Over the past decades, optical sensors based on fluorescent or colorimetric changes have been extensively investigated for the detection of biological analytes due to their non-invasion, simplicity and high sensitivity (Carter et al., 2014; Zhang et al., 2014; Li et al., 2019). Recently, these sensors have also been developed for the diagnosis of AD biomarkers (Zhang and Suslick, 2005; Aliyan, et al., 2019; Yang et al., 2020; Liu et al., 2021; Wang et al., 2021). Fluorescent sensor has been widely applied as a powerful tool for monitoring biomarkers *in vitro* and/or *in vivo* due to its strong signal changes towards targeted analytes. Yi and co-workers reported water-solubility NIR sensors with intramolecular charge transfer that demonstrated fluorescence “turn-on” after selectively binding to A*β* aggregates (Xu et al., 2019). Efforts have been put into imaging biomarkers *in vivo*. Zhu and co-workers successfully designed smart near-infrared (NIR) sensors with aggregation induced emission (AIE) properties for A*β* plaques (Fu et al., 2019). Such sensors employed N,N-dimethylaminophenyl functional moiety as the binding group and modified the structures of electron-donating and hydrophilic moieties, resulting in a NIR sensor with AIE properties. They demonstrated fluorescence “turn-on” towards A*β* aggregates *in vitro*, and exhibit remarkable performance *in situ* mapping of A*β* plaques *in vivo*, such as ultrahigh signal-to-noise ratio, excellent binding affinity to A*β* plaques with blood-brain barrier (BBB) penetrability and outstanding NIR properties, thus providing an alternative approach to in-depth study on protein fibrillogenesis *in vivo*. Additionally, Chang and co-workers developed an A*β* oligomer-selective probe *via* diversity-oriented fluorescence library screening and computational techniques, which showed sensitive and real-time monitoring ability of A*β* oligomer. Interestingly, this sensor demonstrated the ability to cross the BBB and stain A*β* oligomers *in vivo*, and could be considered as a vital imaging agent in AD diagnosis (Teoh et al., 2015). Tau protein sensors for the detection of AD have also received much attention. Kim and others designed BODIPY-based NIR fluorophores for the detection of tau protein associated microtubules, by conjugating and annulating N,N-dimethylaminophenyl functional moieties. The designed sensors with excellent photophysical properties were able to selectively stain tau protein and monitor tau protein aggregate *in vivo* (Verwilst et al., 2017).

Alternatively, colorimetric sensors that exhibit visual changes due to interactions with analytes represent a method of rapid and direct detection of AD biomarkers (Brazaca, et al., 2019). The Xu group described a simply colorimetric sensor based on Au nanoparticles (AuNPs) for the naked-eye detection of amyloid *β*-peptide (Zhou et al., 2015). The sensor utilized color changes of AuNPs induced by alterations of AuNP-A*β* (1–40) triggered aggregation and the coordination of A*β* (1–40) with Cu^2+^ to detect the peptide. The red AuNPs turned purple after interacting with A*β* (1–40) in aqueous solution and then became blue with the further addition of A*β* (1–40) caused by AuNPs aggregation.

A single independent biomarker is often insufficient to reflect AD progression, as demonstrated by the superior AD identification of A*β*42/A*β*40 *vs*. A*β* biomarker alone (Lewczuk et al., 2004). Consequentially, the parallel detection of all amyloid *β* (A*β*) peptide species (monomer, oligomer, fibril) is more valuable than the detection of a single AD biomarker. Lock-and-key sensors based on specific recognition principles perform poorly in the detection of multiple analytes as they were designed to bind selectively to a specific substrate. Therefore, it is of great significance to develop a simple and accurate way to parallel monitor multiple AD biomarkers.

Array-based sensing technologies (synonyms: Chemical tongues or chemical noses) that mimic the mammalian gustatory and olfactory systems have been proven as powerful biological detection and analysis tools, especially for the detection of subtle changes in extremely similar compounds in complex samples (Suslick & Rakow, 2000; H. Lin et al., 2011). In contrast to conventional specific recognition probes, sensor arrays based on pattern recognition can identify multiple analytes simultaneously.

**FIGURE 1:**
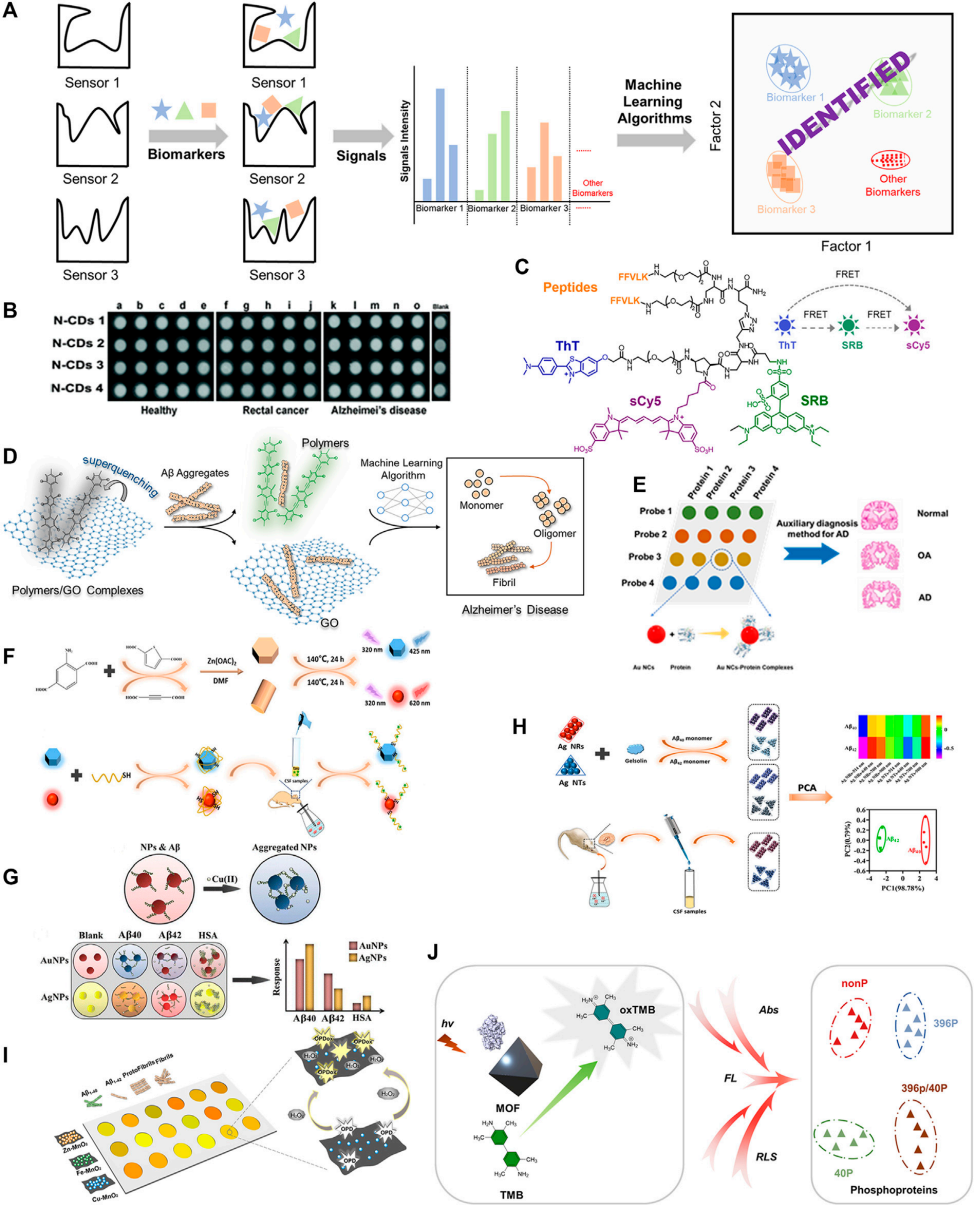
**(A)** Recognition principle of sensor arrays for AD biomarkers. **(B)** The schematic diagram of N-CDs diagnosing AD. Adapted from Xu et al. (2017) with permission. Copyright 2017 Royal Society of Chemistry. **(C)** Molecular structure of combinatorial fluorescent molecular sensor for AD discrimination. Adapted from Hatai et al. (2017) with permission. Copyright 2017 American Chemical Society. **(D)** Design of sensor array constructed by conjugated polymers and graphite oxide electrostatic complexes for the discrimination of Aβ40/Aβ42 aggregates. Adapted from Wang et al. (2022) with permission. Copyright 2022 American Chemical Society. **(E)** The schematic diagram of Au NCs sensor array for proteins discrimination. **(F)** Synthetic method for B- and R-ZnO SMPs and responding mechanism of sensor element towards analytes. Adapted from Liu et al. (2021) with permission. Copyright 2021 American Chemical Society. **(G)** The schematic diagram of the discrimination principle of the colorimetric sensor array. Adapted from Ghasemi et al. (2018) with permission. Copyright 2018 Royal Society of Chemistry. **(H)** The schematic diagram of discrimination principle of Aβ40 and Aβ42 by a colorimetric sensor array based on Ag NTs and Ag NRs. Adapted from Liu et al. (2020) with permission. Copyright 2020 American Chemical Society. **(I)** The schematic diagram of MnO2 nanozyme sensor array detecting Aβ species. **(J)** The schematic diagram of MOF-based multidimensional spectral array for sensitive detection of protein phosphorylation.

Many research endeavors have focused identifying biological samples, such as proteins, saccharides, bacteria, diseases, etc. (Phillips et al., 2008; J. Han et al., 2017; Peveler et al., 2018; Zhu et al., 2021), and recently, sensor arrays for AD core biomarkers detection have also been developed (Kim et al., 2020; Liu et al., 2018). In this review, we will introduce the recent progress of fluorescent/colorimetric sensor arrays, and their applications in the early detection of AD. The associated recognition principles are showed in Figure 1. The summary of optical (fluorescent and colorimetric) sensor arrays for AD diagnosis is shown in Table 1.

**TABLE 1:** Summary of optical sensor array for AD diagnose.

Sensor array	Sensor array type	Test biomarkers	Test concentration	Clinical samples	References
1	Fluorescent signal	Protein	100 mg/ml	Yes	Xu et al. (2017)
2	Fluorescent signal	A*β*40, A*β*42	30 μM	No	Hatai et al. (2017)
3	Fluorescent signal	A*β*40, A*β*42	5.0 µM	No	Wang et al. (2022)
4	Fluorescent signal	Protein	1 mg/ml	Yes	Han et al. (2019)
5	Fluorescent signal	Tau 381, Tau 410, Tau 441	500 pg/ml	Rats model	Liu et al. (2021)
6	Colorimetric signal	A*β*40, A*β*42	300 nM	NO	Ghasemi et al. (2018)
7	Colorimetric signal	A*β*40, A*β*42	300 nM	Rats model	Liu et al. (2020)
8	Colorimetric signal	A*β*40, A*β*42	0.1 nM	Yes	Hu et al. (2022)
9	Multidimensional signal	Protein phosphorylation	400 nM	NO	Lin et al. (2021)

## Fluorescent Sensor Array

Compared with other analysis techniques, the fluorescence-based detection technique is one of the most powerful approaches for biological and environmental analysis owing to its selectivity and sensitivity (Chen et al., 2016; Balamurugan et al., 2018; Wang et al., 2021). The Luo group described a simple approach to construct a label-free fluorescent sensor array based on four nitrogen-doped carbon dots (N-CDs) to achieve simultaneous detection of multiple proteins, as shown in Figure 1B (Xu et al., 2017). Different surface properties of the four N-CDs induced distinctive affinities with multiple proteins and further resulted in the distinctive fluorescence responses, enabling the qualitative and quantitative discrimination of various proteins. They also tried such an array for discriminating among the serums of rectal cancer patients, AD patients and healthy people, and they were successful (Figure 1B). The label-free strategy demonstrated a simple way to build sensor arrays for the detection of analytes, however, the sensitivity of sensor array for detection may be seriously affected non-specific effects and makes it difficult to meet the requirement of detection.

The Förster resonance energy transfer (FRET) principle is a rational and effective design strategy for constructing sensor arrays. Margulies et al described a combinatorial fluorescent molecular sensor based on the FRET principle (Hatai et al., 2017). In this approach, three fluorescent reporters with multiple binding sites were assembled into a single sensor. By introducing two site-specific recognizing groups, bis-KLVFF peptide and thioflavin T (ThT), the engineered sensor was capable of generating diverse fluorescence responses to various A*β* aggregates (low molecular weight oligomers, high molecular weight oligomers, protofibrils, and fibrils.), as shown in Figure 1C. Three fluorescence reporters (ThT, SRB, sulfo-Cy5) successfully generated fingerprints of A*β* aggregates. The combination strategy reduces the number of sensors required for diagnosis, thus reducing workload, but synthesis of the sensing elements can be highly complex. Overall, by utilizing the intramolecular FRET approach for sensor design and the incorporation of specific/non-specific recognition and fluorescent reporters, the recognition efficiency of amyloid-beta aggregates can be greatly enhanced.

Compared with small molecular fluorescent sensors, conjugated polymers generate an amplified fluorescent response *via* external structural perturbations and electron-density changes of the delocalizable *π*-electron polymer chain backbone after reacting with a targeted analyte (Bunz, et al., 2015). Recently, our group has developed a hypothesis-free fluorescent sensor array composed of electrostatic complexes formed from five positively charged poly (*para*-aryleneethynylene) (PAEs) and one negatively charged graphite oxide (GO) (Wang et al., 2022). The fluorescence of PAEs was strongly quenched by GO with high binding constants (K_*SV*_ range from 10^13^ to 10^15^ M^−1^). Different types of A*β* aggregates and various concentrations of A*β*40/A*β*42 could be distinguished by the sensor array formed by the electrostatic complexes with 100% accuracy *via* optimized machine learning algorithms, indicating significant potential for early diagnosis of Alzheimer’s disease (Figure 1D). In cases that require high sensitivity of the sensor array due to low sample concentration, fluorescent sensor arrays based on conjugated polymers might be a good choice.

A gold nanocluster-based fluorescence sensor array for AD diagnosis was developed by Li’s group (Han et al., 2019). They designed and synthesized four gold nanoclusters modified with THPC/GSH, BSA, His/MU and DTT to generate different surface properties and fluorescence behaviors in response to A*β* biomarkers/tau proteins (Figure 1E). Protein discrimination was achieved *via* pattern recognition with linear discriminant analysis. Furthermore, serum of AD patients, osteoarthritis patients and healthy people could be successfully discriminated by this array. Overall, the simple modification method of the gold nanoclusters and successful identification of AD patents from other conditions demonstrate the significant potential of this fluorescent sensor.

Deng’s group reported a sensor array consisting of blue fluorescence ZnO submicron particles (B-ZnO SMPs) and red fluorescence ZnO submicron particles (R-ZnO SMPs) to target tau proteins. The array was constructed by the coordination of Zn^2+^ with carboxyl groups of organic ligands of various structures were used to detect AD upon Cd^2+^ exposure (Liu et al., 2021). The surface of ZnO submicron particles were coated with tau aptamer through Zn-S bond and electrostatic adsorption to selectively combine with tau species. The examined fluorescence changes were triggered by the bonding of tau protein species and subsequent surface morphology transformations (Figure 1F). B-ZnO SMPs and R-ZnO SMPs showed differential fluorescence changes towards tau381 (3R tau isoform), tau410 (3R tau isoform) and tau441 (4R tau isoform) resulting from varying tau aptamer-isoform bond strengths (based on genetic sequences) and SMP-isoform interactions. The fluorescence data was collected and used to form fingerprints for qualitative and quantitative analysis of the above tau isoforms. More importantly, this sensor array allowed monitoring of tau441% changes [tau441/(tau441 + tau381)] in the cerebrospinal fluid, demonstrating its potential to monitor the 4R tau:3R tau ratio for the AD early diagnosis, as the 4R:3R ratio is believed to play a vital role in the formation of neurofibrillary tangles. Thus, the conjugated SMP approach outlined, making use of specific sensor aptamers, demonstrates a rational construction methodology for AD sensor arrays and excellent potential for diagnosis.

## Colorimetric Sensor Array

Colorimetric detection techniques have attracted great attention due to their prominent advantages, such as naked-eye recognition, low cost, simple operation, non-specialist equipment, real-time detection, as well as high sensitivity and selectivity towards various analytes (Abedalwafa et al., 2019). A label-free sensor array for diagnosing AD *via* monitoring *β*-amyloid peptides was developed by Mahmoudi and others (Figure 1G) (Ghasemi, et al., 2018). A*β*40, A*β*42 and HSA showed various affinities with AuNPs/AgNPs. The addition of Cu(II) ions led to variations in the AuNPs/AgNPs aggregates, which eventually produced colorimetric changes. This colorimetric sensor array demonstrated excellent ability to detect A*β*40 and A*β*42 in the absence/presence of interference or human plasma. The colorimetric change derived from the changes of nanoparticle aggregates that affected by analyte offers an alternative strategy for the discrimination of targeted analyte.

Zhou and co-workers modified silver nanotriangles (Ag NTs) and silver nanorods (Ag NRs) with gelsolin and examined the colorimetric and spectral changes induced by the aggregations of Ag NTs and Ag NRs for monitoring the CSF A*β*42% [A*β*42/(A*β*40+A*β*42)], see Figure 1H (Liu et al., 2020). The authors selected the multichannel absorption intensities at 514 nm, 640 nm, 700 nm, and 900 nm to construct a unique fingerprint for each analyte *via* principal component analysis. The NP sensor array successfully discriminated between A*β*40 and A*β*42, and was capable of detecting A*β*42% in complex CSF samples owing to the high selectivity.

The limit of detection (LOD) for analytes is a key indicator for biological analysis, determining whether the probe can meet practical demands. Therefore, an approach with high LOD is required. The Luo group developed a nanozyme sensor array based on metal (Zn/Fe/Cu)-manganese dioxide (MnO_2_) for distinguishing between A*β* species with an excellent LOD. As shown in Figure 1I, it was even used to monitor the formation of A*β* aggregates. More importantly, the nanozyme sensor array could discriminate between AD patents and healthy individuals clinical blood samples with 85% accuracy (Hu et al., 2022). The authors reported that the nanozyme metal-MnO_2_ played a peroxidase-mimicking role in the oxidation from o-phenylenediamine (OPD, colorless) to oxidized OPD (OPDox, yellow) in the presence of hydrogen peroxide. The varied affinities of A*β* species towards Zn/Fe/Cu-MnO_2_ resulted in distinctively modulated peroxidase-mimicking activity, leading to the diverse color changes from various oxidation of OPD, which were employed for pattern recognition. As demonstrated by the Luo group, enzyme and enzyme-mimetic systems are excellent methods to generate high sensitivity sensor arrays towards analytes, and demonstrate the possibility of a clinical diagnostic systems for detecting AD. These nanozyme-like systems could open a wide range of future research avenues in biomedical fields and lead to detection arrays for numerous other diseases.

## Multiple Spectral Sensor Array

The Chen group reported a simple sensor array constructed from a multifunctional metal-organic framework (MOF) for the detection of phosphoproteins, see Figure 1J (Lin, et al., 2021). Differing from other analytical methods, the team built a multi-signal spectral sensor array [absorbance, fluorescence and resonance light scattering (RLS)] instead of a multi-element sensor array or congeneric multi-channel sensor array. The spectral changes were induced by the adsorption of phosphoproteins on the surface of MOF hybrid and could identify different phosphoproteins with high sensitivity, demonstrating its potential in distinguishing tau peptides or phosphoprotein-related diseases through a multidimensional signal’s sensor array. Multiple spectral sensor arrays have the advantage of reduced numbers of sensor elements and improved efficiency of detection. Further research and development into biomolecule detection, coupled with the reduced components and increase efficiency, could make this sensor array approach a research hotspot.

## Summary and Perspective

In this review, we focus on the recent advances of the optical sensor array for the parallel discrimination of key biomarkers of AD and clinical samples of AD patients, including fluorescent sensor arrays, colorimetric sensor arrays and multiple spectral sensor arrays. Although little research has been reported on the applications of optical sensor arrays for the early diagnosis of AD, these efforts appear to be an extremely promising avenue compared to the previously reported detection methods for single AD biomarkers. Meanwhile, there are still significant debates regarding on the positive correlation between A*β* peptides/tau proteins and AD phenotype, indicating the identification of AD phenotype by specific probes of A*β* peptide or tau protein is still challenging. Sensor array based on non-specific principle can reflect holistic differences through cross-reactive signals, just as the five or six taste receptors of human tongue can recognize thousands of flavors, and thus it can detect AD phenotype *via* pattern recognition. We firmly believe that optical sensor arrays for early AD diagnosis should receive more attention due to their disease detection potential and can greatly accelerate clinical diagnosis. The key steps to achieving this should be: 1) The focus should be on improving the detection limits of AD biomarkers, 2) reducing the number of sensor elements, 3) improving detection efficiency, 4) reducing detection costs of sensor arrays. As shown in Luo’s work, optical multichannel signals of one sensor element and heterogeneous multidimensional signals of the sensor array, as well as dynamical combinational chemistry strategies, are potentially powerful tools to reduce the workload *via* decreasing sensor elements. As well, enzyme and enzyme-mimetic systems can tremendously improve the sensitivity of sensor arrays, including enzyme cascade amplification strategy. Overall, colorimetric and fluorescent optical sensor arrays have phenomenal potential for further biomedical development and will be a fundamental health screening and monitoring systems for AD, and numerous other diseases, in the future.
